# primerJinn: a tool for rationally designing multiplex PCR primer sets for amplicon sequencing and performing in silico PCR

**DOI:** 10.1186/s12859-023-05609-1

**Published:** 2023-12-12

**Authors:** Jason D. Limberis, John Z. Metcalfe

**Affiliations:** Division of Pulmonary and Critical Care Medicine, Trauma Centre, Zuckerberg San Francisco General Hospital, University of California, San Francisco, San Francisco, CA USA

**Keywords:** PCR, Primer design, Targeted sequencing, Multiplex PCR

## Abstract

**Background:**

Multiplex PCR amplifies numerous targets in a single tube reaction and is essential in molecular biology and clinical diagnostics. One of its most important applications is in the targeted sequencing of pathogens. Despite this importance, few tools are available for designing multiplex primers.

**Results:**

We developed primerJinn, a tool that designs a set of multiplex primers and allows for the in silico PCR evaluation of primer sets against numerous input genomes. We used primerJinn to create a multiplex PCR for the sequencing of drug resistance-conferring gene regions from *Mycobacterium tuberculosis*, which were then successfully sequenced.

**Conclusions:**

primerJinn provides a user-friendly, efficient, and accurate method for designing multiplex PCR primers for targeted sequencing and performing in silico PCR. It can be used for various applications in molecular biology and bioinformatics research, including the design of assays for amplifying and sequencing drug-resistance-conferring regions in important pathogens.

## Introduction

Multiplex PCR amplifies numerous targets in a single tube reaction and is essential in molecular biology and clinical diagnostics. One of its most important applications is in the targeted sequencing of pathogens. By using multiple primer pairs to amplify specific target regions in a single reaction, multiplex PCR allows for the simultaneous detection and identification of multiple pathogens or drug resistance-conferring regions in a single sample, making it a valuable tool for diagnostic and epidemiological studies. Despite this importance, few tools are available for designing multiplex PCR primers suitable for targeted sequencing, [1–7] some of which are no longer accessible, and none of which also perform in silico PCR. This is because designing primers for multiple targets simultaneously is challenging, requiring careful consideration of multiple factors such as primer specificity, to reduce off target amplification; amplicon length, to ensure relatively even proportions of amplicons; and primer interactions, to limit primer dimer formation, under the specific reaction conditions for the, typically high-fidelity, polymerase needed for accurate sequencing, which require higher annealing temperatures. Yet, these parameters are critical for designing efficient primer sets for use on clinical samples, which may have low amounts of DNA and contain numerous different microbes.

We developed primerJinn, a tool that designs a set of multiplex PCR primers and allows for the in silico PCR to evaluate them against numerous input genomes. primerJinn uses primer3 [8] to create primers and a clustering method to select the best primer set based on the amplicon size, melting temperature, and primer interactions. The in silico PCR function uses BLAST [9] to identify primer pairs that amplify a DNA sequence of a user-specified maximum, at a given annealing temperature and salt concentration and provides detailed information about the primers and amplicons. Our tool also incorporates approximations for melting temperatures utilizing Q5 Hot Start High-Fidelity Polymerase buffers (NEB, USA), which differ significantly from most other polymerases. primerJinn provides an efficient and accurate method for designing multiplex PCR primers and performing in silico PCR and can be a valuable resource for researchers in the field of targeted sequencing for pathogens.

## Implementation

### Primer design

**Fig. 1:**
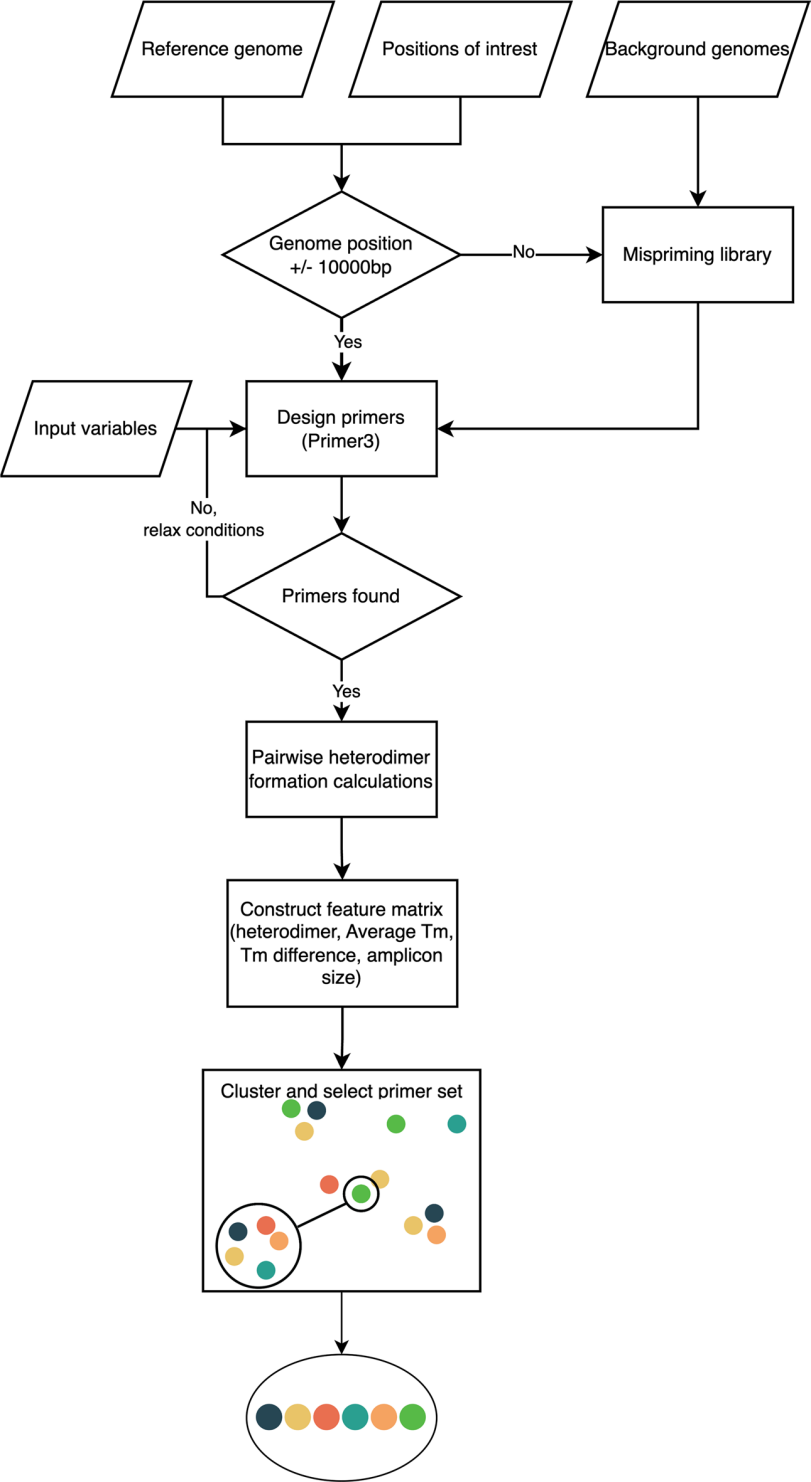
primerJinn primer design workflow

primerJinn is written in Python and uses primer3 to design primers surrounding each specific target range in an input design FASTA file (Fig. 1). By default, these primers amplify a region of 400–800 nucleotides, have an optimal length of 20 (range 10, 40) nucleotides, an optimal Tm of 65 °C (range 62 °C, 68 °C). These are optimal for amplification with a high-fidelity polymerase and subsequence sequencing using Illumina. Primers are checked for specificity against a mispriming library consisting of an optional input background FASTA file, and the portions of the input design FASTA file that are not used to design a particular primer pair. If no primer pair can be designed for a target, the input parameters are iteratively relaxed until a pair is found. Since high-fidelity polymerase buffers tend to increase the Tm of primers, we have included an approximation for the highest-fidelity polymerase, Q5 from NEB. We also use the NEB Tm calculator API to output the final Tm for the selected primer set. Following the design of one hundred (default value) primer pairs for each region, a matrix is constructed including Tm and amplicon size for each primer pair, and heterodimer formation probability (based on the Gibbs free energy) for each primer combination. This matrix is used to generate clusters. The cluster with the most regions covered is selected, and missing primers are added from the next closest cluster using an Euclidean metric and Ward linkage criterion. The output is written to an Excel file, and includes Illumina adapter tails if selected, allowing for the nested PCR barcoding of samples.

### In silico PCR

primerJinn also allows for the in silico PCR evaluation of primers. It takes a reference FASTA file and primer sequences as input and returns the binding position (located using blastn-short) and product length of any pair of primers that generate a product at, or below the input Tm (default is 70 °C) and the maximum amplicon size (default is 2000 nucleotides). Options include annealing temperature, salt concentration, maximum product length, and whether two or more bases at 5′ end of the primer are required to bind. The output is written to an Excel file.

## Results

**Fig. 2:**
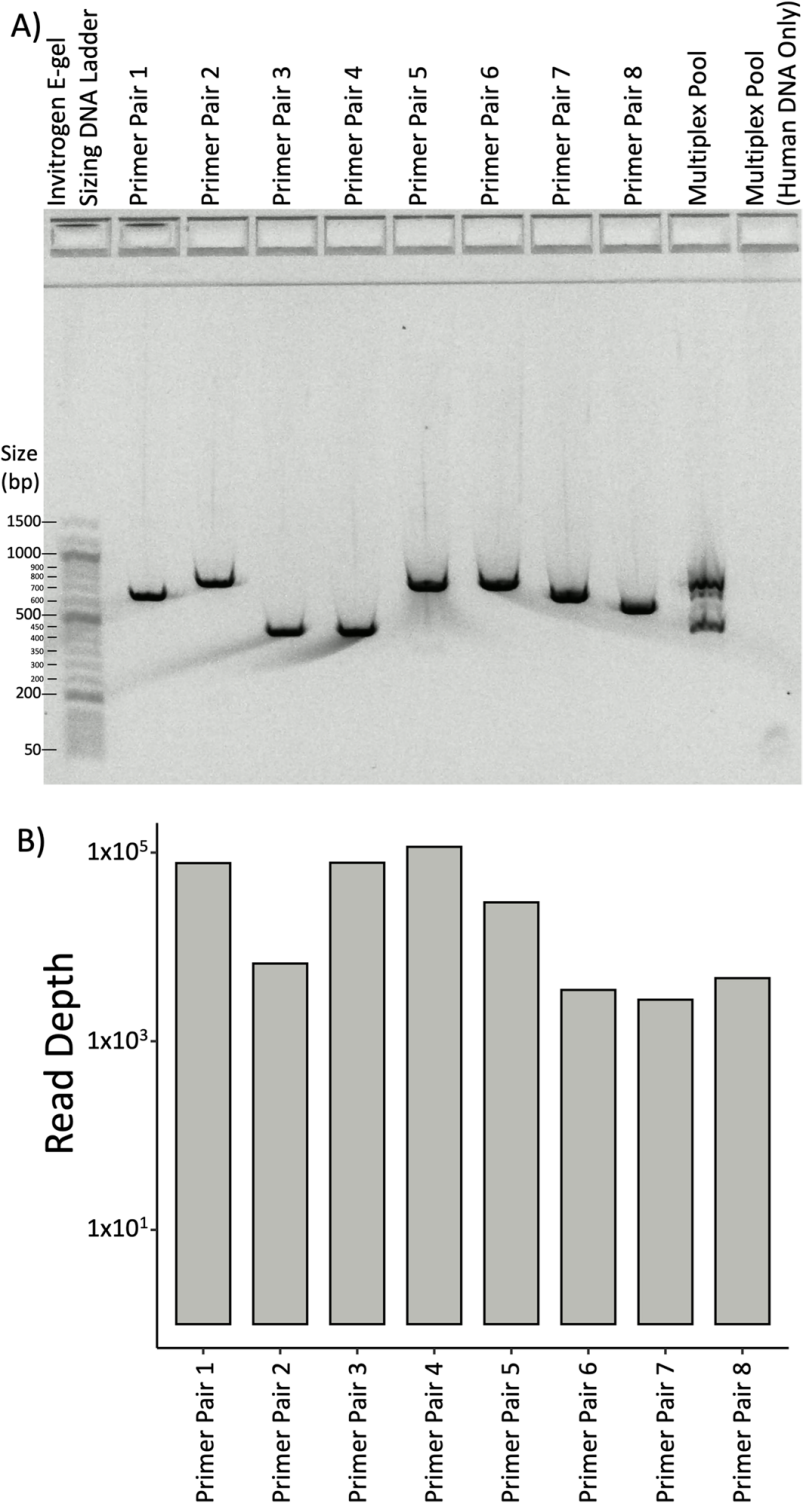
**A** Electrophoresis gel showing expected amplicon sizes and specificity for each individual and pooled primer pairs and **B** coverage of the sequencing of the pooled multiplex PCR from lane 8

To evaluate primerJinn we selected eight drug resistance-conferring gene regions from *Mycobacterium tuberculosis*, the etiological agent of tuberculosis. We passed the 4.4 Mb, high GC (~ 65%) genome FASTA (NC_000962.3) to primerJinn with the regions listed in Table 1. primerJinn output one primer set for each position (Table 2), with the mean primer Tm and amplicon size being 65 °C (range 64 °C, 67 °C) and 665 nucleotides (range 454, 791), respectively. We then used these primers as input for primerJinn in silico PCR function, which appropriately returned only the eight expected amplicons. Finally, we synthesized the 16 primers and performed singleplex and multiplex PCR on *M. tuberculosis* H37Rv genomic DNA using NEB Q5 HotStart DNA polymerase MasterMix for 35 cycles with denaturation, annealing, and extension for 10s at 98 °C, 20s at 65 °C and 30s at 72 °C. We ran electrophoresis gels and saw the expected bands (Fig. 2A), which showed no mispriming to the human genome (lane 10). We then sequenced the amplicon pool using 2 × 250 bp paired-end Illumina sequencing and aligned the reads to the target amplicons using BWA-men [10]. We had an 82% alignment to the target amplicons (Fig. 2B) at similar depths for each amplicon, with an unaligned remainder consisting of low-quality reads, singletons, and primer dimers. We also evaluated our Q5 Tm approximation settings against 10,000 random DNA sequences from 15 to 25 nucleotides long (1000 in each group). Our Tm approximations were a mean of − 0.21 °C (SD 0.71 °C) below that of the NEB Tm.

**Table 1:** Genomic targets for primerJinn multiplex design of drug resistance-conferring regions on *M. tuberculosis* H37Rv (NC_000962.3)

Gene	Drug	Genomic position start	Genomic position end
*gyrB*	Fluoroquinolones	6578	7250
*fgd1*	Delamanid	490,900	491,416
*rpoB*	Rifampicin	761,007	761,277
*rv0678*	Bedaquiline	778,990	779,488
*fbiC*	Linezolid	1,303,831	1,303,911
*atpE*	Bedaquiline	1,461,045	1,461,291
*inhA*	Isoniazid	1,674,182	1,674,222
*katG*	Isoniazid	2,154,831	2,154,873
*pncA*	Pyrazinamide	2,288,681	2,289,242

**Table 2:** primerJinn output for eight drug resistance conferring gene regions of *M. tuberculosis* H37Rv (NC_000962.3)

Primer pair	Forward primer (5′->3′)	Reverse primer (5′->3′)	Forward Tm (°C)	Reverse Tm (°C	Product Size (bp)	Name	Forward Primer Tm NEB (°C)	Reverse Primer Tm NEB (°C)
1	GAGGAACACCACTAGTACCG	CTCGATGACTTTACGGCCAT	65	64	675	Target 1,461,045–1,461,291	64	64
2	CATGGGATATGGAGCGATCG	GGGGTCGTAGGAGATCTTGA	66	66	791	Target 490,900–491,416	65	65
3	CCGGTTGTCCATTCCGTTTA	CTGTACGTATTTGGGTTGCG	64	66	454	Target 1,303,831–1,303,911	65	64
4	GGATGCGAGCTATATCTCCG	AATACGCCGAGATGTGGATG	65	65	458	Target 1,674,182–1,674,222	65	65
5	CAACAGTTCATCCCGGTTCG	GACGGATTTGTCGCTCACTA	65	67	759	Target 2,288,681–2,289,242	66	64
6	GCCACCATCGAATATCTGGT	GCTCCAGGAAGGGAATCATC	66	65	778	Target 761,007–761,277	64	65
7	GCATACCGAACGTCACAGAT	ACGGTCACCTACAAAAACGG	66	65	665	Target 778,990–779,488	65	65
8	GCTCTTAAGGCTGGCAATCT	CGGTCACACTTTCGGTAAGA	65	66	577	Target 2,154,831–2,154,873	65	64

Annealing temperatures (Tms) are in Celsius (Tms have been rounded for illustration purposes), and the product sizes are given in nucleotides

## Discussion

primerJinn provides a user-friendly, efficient, and accurate method for designing multiplex PCR primers and performing in silico PCR. We demonstrate that primerJinn can design a multiplex PCR primer for a reaction using Q5 high-fidelity polymerase for an Illumina targeted sequencing and generate similar coverage per target. There are several limitations to primerJinn. First, primerJinn requires the user to input the regions of interest and cannot fix any overlapping regions in this list. Second, it is only designed for designing primers for sequencing and cannot design primers producing different amplicon sizes for end-point PCR bases analysis, nor can it design primer probe pairs for qPCR or degenerate primers. Finally, it does not have a graphical user interface, which could be a barrier for some users. primerJinn can be used for various applications in molecular biology and bioinformatics research, including the design of assays for amplifying and sequencing drug-resistant conferring regions in important pathogens.

## Conclusion

primerJinn provides a user-friendly, efficient, and accurate method for designing multiplex PCR primers for targeted sequencing and performing in silico PCR.

## Availability and requirements

Project name: primerJinn. Project home page: https://github.com/SemiQuant/PrimerJinn. Operating system(s): Platform independent. Programming language: Python. Other requirements: BLAST+. License: GNU GPL.

Any restrictions to use by non-academics: License needed.

## Data Availability

Code and raw data are available at https://github.com/SemiQuant/PrimerJinn.
